# Supplementary material for: Gas Chromatography–Mass Spectrometry Profiling of Volatile Compounds Reveals Metabolic Changes in a Non-Aflatoxigenic Aspergillus flavus Induced by 5-Azacytidine
Source: Toxins (Basel). 2020 Jan 19;12(1):57. doi: 10.3390/toxins12010057 (PMC7020457; doi:10.3390/toxins12010057)
Supplement: Supplementary file 1 [file toxins-12-00057-s001.pdf]

Supplementary Materials: Gas Chromatography–Mass Spectrometry Profiling of Volatile Compounds Reveals Metabolic Changes in a Non-Aflatoxigenic *Aspergillus flavus* Induced by 5-Azacytidine

Fengqin Song, Qingru Geng, Xuewei Wang, Xiaoqing Gao, Xiaona He, Wei Zhao, Huahui Lan, Jun Tian, Kunlong Yang and Shihua Wang

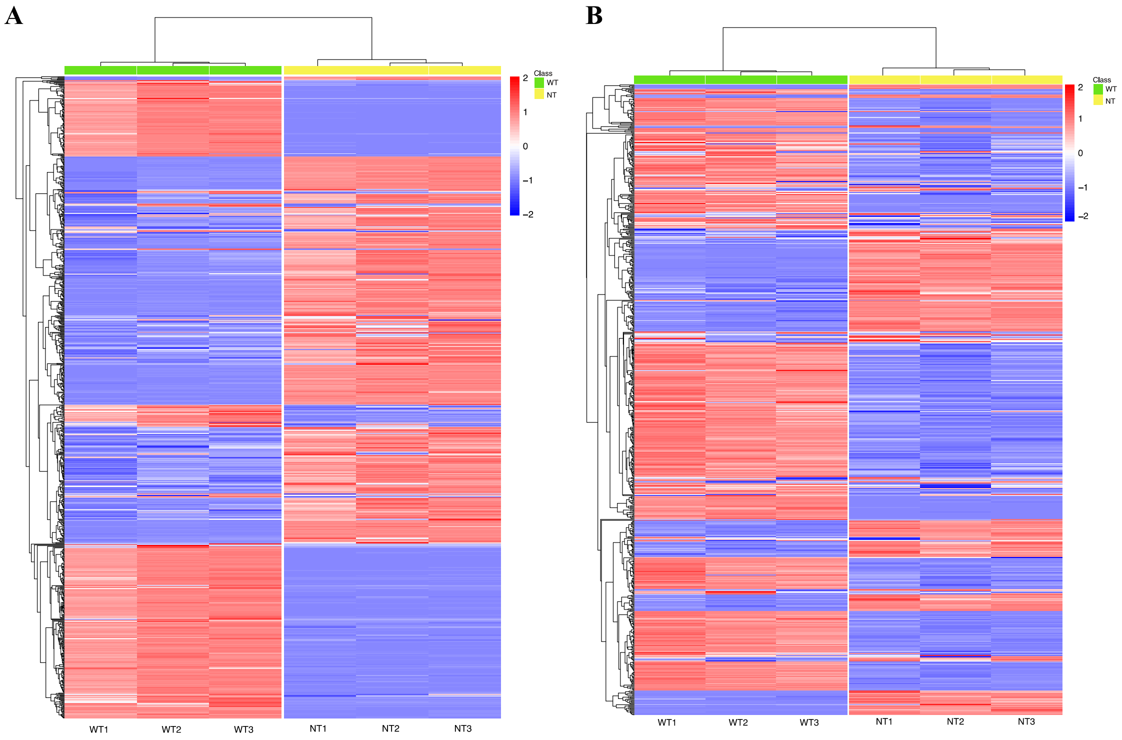

Figure S1. Clustering and heatmap visualization of the identified volatile metabolites in WT and NT strains both in vitro and in vivo. (**A**) Clustering and heatmap visualization of the identified volatile metabolites in WT (A133 strain) and NT strains in vitro. (**B**) Clustering and heatmap visualization of the identified volatile metabolites in WT and NT strains in vivo.

Table S1. Volatile metabolites assessed via GC-MS analysis in WT (A133 strain) and NT strains in vitro were analyzed with the Agilent ChemStation software. The chemical structure or the chemical structural formula was analyzed in pubchem database (https://pubchem.ncbi.nlm.nih.gov).

RT (min)	Compound Name	Quality (%)	mz	CAS Number	Structure	Formula	WT	NT
				**Fatty Acid Derived Volatiles**				
19.361	Ethanol,2-(2-butoxyethoxy)-	90	162.126	000112-34-5	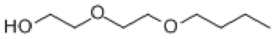	$C_8H_{18}O_3$	Y	Y
28.330	Dimethylphthalate	91	194.058	000131-11-3	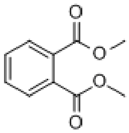	$C_{10}H_{10}O_4$	Y	Y
39.928	Phthalicacid,isobutylnonylester	90	348.23	003461-31-2	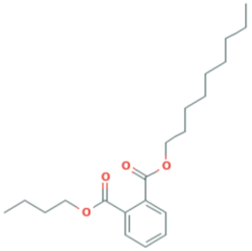	$C_{21}H_{32}O_4$	Y	N
40.499	Allyl isovalerate	30	142.099	002833-93-4	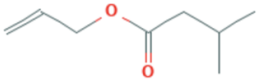	$C_8H_{14}O_2$	Y	N
41.000	7,9-Di-tert-butyl-1-oxaspiro(4,5)deca-6,9-diene-2,8-dione	99	276.173	082304-66-3	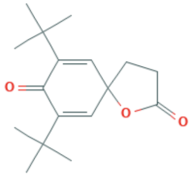	$C_{17}H_{24}O_3$	Y	Y
41.700	Pentadecanoicacid,14-methyl-,methylester	74	270.256	005129-60-2	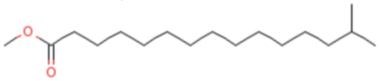	$C_{17}H_{34}O_2$	Y	Y

42.542	n-Hexadecanoicacid	93	256.24	000057-10-3	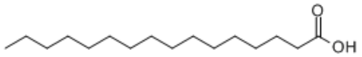	$C_{16}H_{32}O_2$	Y	N
42.996	Tetradecanoic acid	93	228.209	000544-63-8	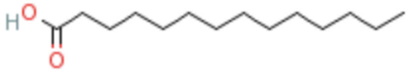	$C_{14}H_{28}O_2$	Y	N
43.325	Hexadecanoicacid,ethylester	98	284.272	000628-97-7	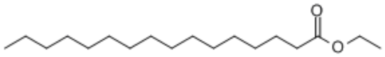	$C_{18}H_{36}O_2$	Y	N
44.343	Normeperidinic acid	59	205	003627-45-0	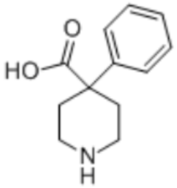	$C_{12}H_{15}NO_2$	Y	Y
45.441	1,4-Naphthoquinone,6-ethyl-2,5-dihydroxy-	38	218.058	013378-87-5	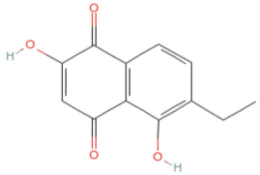	$C_{12}H_{10}O_4$	Y	N
45.600	9,12-Octadecadienoicacid(Z,Z)-,methylester	99	294.256	000112-63-0	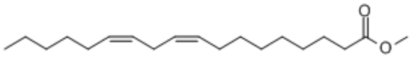	$C_{19}H_{34}O_2$	Y	N
46.496	9,12-Octadecadienoicacid(Z,Z)-	99	280.24	000060-33-3	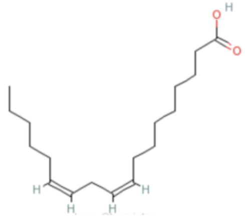	$C_{18}H_{32}O_2$	Y	N
46.628	6-Octadecenoicacid	96	282.256	1000336-66-8	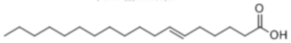	$C_{18}H_{34}O_2$	Y	N
47.111	9,12-Octadecadienoicacid,ethylester	99	308.272	007619-08-1	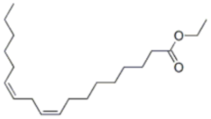	$C_{20}H_{36}O_2$	Y	N

47.272	n-Propyl9-octadecenoate	64	324.303	1000336-71-6	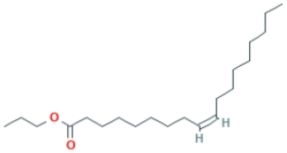	$C_{21}H_{40}O_2$	Y	N
47.660	OleylAlcohol	90	268.277	000143-28-2	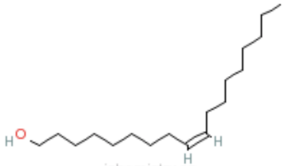	$C_{18}H_{36}O$	Y	N
47.894	Heptadecanoicacid,15-methyl-,ethylester	94	312.303	057274-46-1	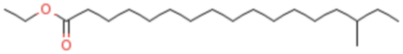	$C_{20}H_{40}O_2$	Y	N
51.116	Z,E-7,11-Hexadecadien-1-yl acetate	51	280.24	051607-94-4	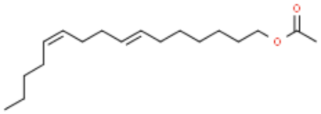	$C_{18}H_{32}O_2$	N	Y
51.951	Hexanedioicacid,bis(2-ethylhexyl)ester	93	370.308	000103-23-1	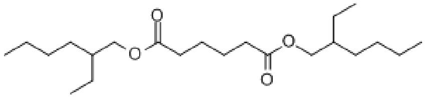	$C_{22}H_{42}O_4$	Y	N
52.434	E-8-Methyl-9-tetradecen-1-ol acetate	38	268.24	1000130-81-4	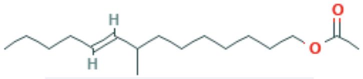	$C_{17}H_{32}O_2$	Y	N
60.276	6,9-hexadecadienoic acid	55	252.39	1000253-06-3	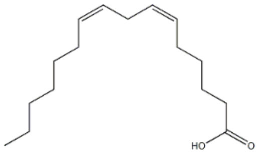	$C_{16}H_{28}O_2$	Y	N

66.434	Ergosterol	94	396.339	000057-87-4	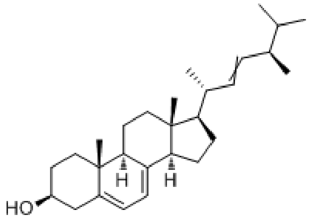	$C_{28}H_{44}O$	Y	N
				**Saccharide Derived Volatiles**				
34.400	Benzo[b]tetrahydrofuran-3-one,5,6-dihydroxy-	59	166.027	014771-00-7	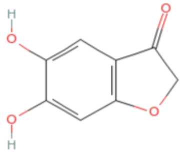	$C_8H_6O_4$	Y	Y
53.474	3-Methyl-2-(2-oxopropyl)furan	49	138.068	087773-62-4	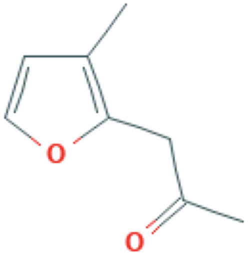	$C_8H_{10}O_2$	Y	N
				**Amino Acids Derived Volatiles**				
6.203	2-Propenamide	72	71.037	000079-06-1	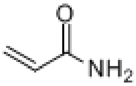	C_3H_5NO	N	Y

29.333	N-Methyl-N-methoxy-5,6,7,8-tetrahydro-1-naphtamide	64	219.126	185957-97-5	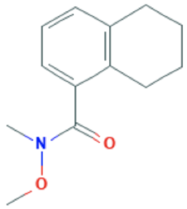	$C_{13}H_{17}NO_2$	Y	N
29.700	Caulophylline	86	204.27	000486-86-2	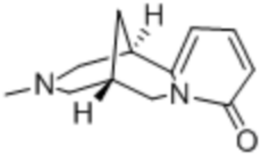	$C_{12}H_{16}N_2O$	Y	Y
31.903	Pyrazine,2-methoxy-3-(1-methylethyl)-	49	152.095	025773-40-4	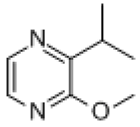	$C_8H_{12}N_2O$	N	Y
33.200	L-Tyrosine	94	182	000060-18-4	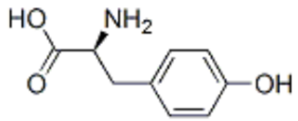	$C_{18}H_{32}O$	Y	Y
33.206	Piperonylamine	38	151.063	002620-50-0	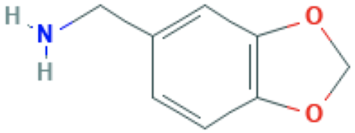	$C_8H_9NO_2$	N	Y

33.895	4'-Azidobenzo[1',2'-b]-1,4-diazabicyclo[2.2.2]octene	25	201.101	120287-75-4	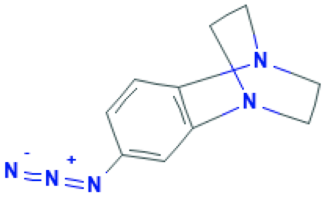	$C_{10}H_{11}N_5$	Y	N
34.927	l-Alanine,N-(3-fluorobenzoyl)-,pentylester	64	281.143	1000314-20-8	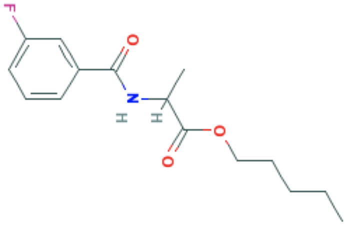	$C_{15}H_{20}FNO_3$	Y	N
35.022	2,6-toluenediamine	59	122.17	000823-40-5	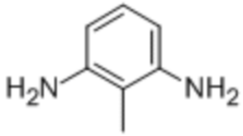	$C_7H_{10}N_2$	N	Y
35.930	6-Methyl-3,5-heptadien-2-one	91	124.18	001921-70-6	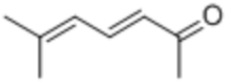	$C_8H_{12}O$	N	Y
36.911	Theobromine	87	180.167	000083-67-0	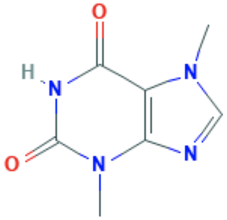	$C_7H_8N_4O_2$	Y	N

38.317	4-Amino-9-fluorenone	46	195.068	004269-15-2	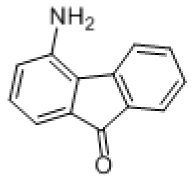	$C_{13}H_9NO$	Y	N
41.019	d-Proline, N-allyloxycarbonyl-, heptadecyl ester	43	437.351	1000320-97-6	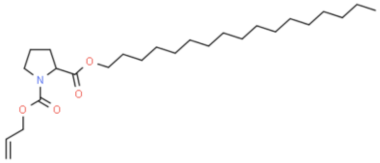	$C_{26}H_{47}NO_4$	N	Y
41.180	3-Cyclohexyloxypropylamine, N,N-dimethyl-	50	185.178	071126-67-5	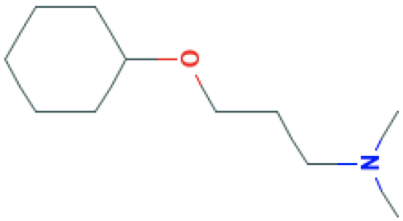	$C_{11}H_{23}NO$	N	Y
41.253	Sulfurous acid, 2-ethylhexyl isohexyl ester	38	278.192	1000309-19-0	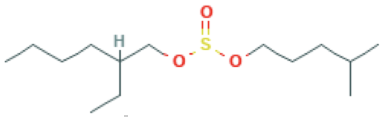	$C_{27}H_{56}$	Y	Y
41.480	Pyrrolo[1,2-a]pyrazine-1,4-dione,hexahydro-3-(2-methylpropyl)-	64	210.137	005654-86-4	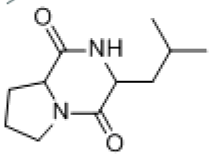	$C_{11}H_{18}N_2O_2$	N	Y

41.500	Diethyldithiophosphinicacid	38	154.004	000866-54-6	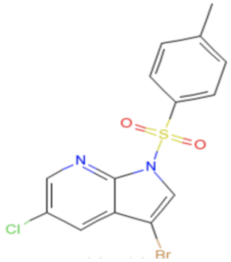	$C_{14}H_{10}BrClN_2O_2S$	Y	Y
41.663	Phenylacetylglycine	27	193.202	000500-98-1	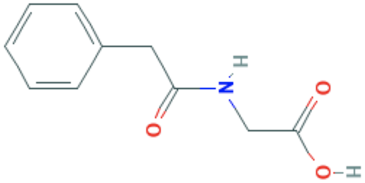	$C_{10}H_{11}NO_3$	N	Y
46.210	N-Methyl-beta-carboline-3-carboxamide	94	225.25	078538-74-6	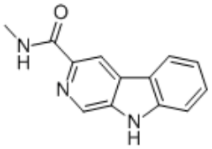	$C_{13}H_{11}N_3O$	N	Y
47.418	Hexadecanamide	97	255.256	000629-54-9	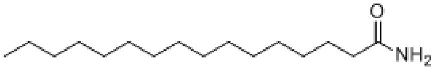	$C_{16}H_{33}NO$	N	Y
47.455	9-Octadecenamide,(Z)-	95	281.272	000301-02-0	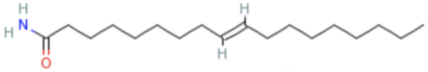	$C_{18}H_{35}NO$	Y	N
49.747	4-(3,4-Dihydro-2H-quinolin-1-yl)-4-oxo-butyric acid hydrazide	43	247.132	315673-61-1	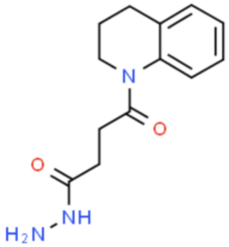	$C_{13}H_{17}N_3O_2$	Y	N

50.040	Tris(aziridinomethyl)hydrazine	58	197.164	1000254-64-1	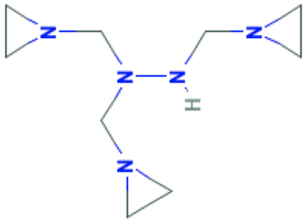	$C_{34}H_{70}$	Y	N
50.413	Pyrrolo[1,2-a]pyrazine-1,4-dione,hexahydro-3-(phenylmethyl)-	49	244.121	014705-60-3	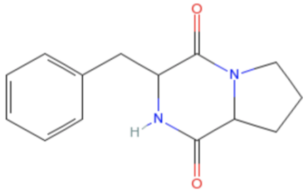	$C_{14}H_{16}N_2O_2$	N	Y
51.270	Heptanamide, 4-ethyl-5-methyl-	42	171.162	054789-40-1	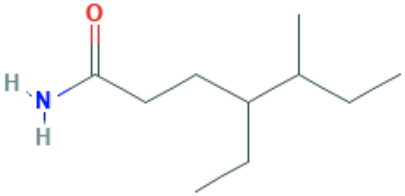	$C_{10}H_{21}NO$	Y	Y
53.298	Cyclohexene,6-butyl-1-nitro-	46	183.126	084820-13-3	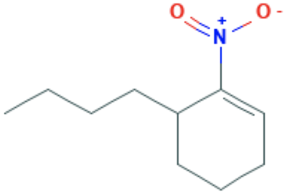	$C_{10}H_{17}NO_2$	Y	N
54.404	4-Morpholineethanol	46	131.095	000622-40-2	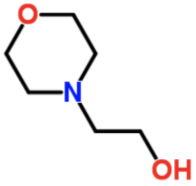	$C_6H_{13}NO_2$	Y	N

54.887	1-(2-Thiazolylazo)-2-naphthol	81	255.5	001147-56-4	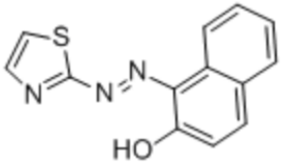	$C_{13}H_9N_3OS$	Y	N
55.487	Trifluoroacetic acid,n-tridecyl ester	38	296.196	053800-02-5	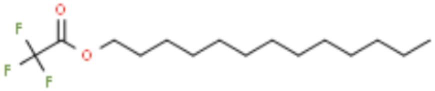	$C_{15}H_{27}F$	Y	N
57.450	1H-Indole,4-methyl-	43	131.073	016096-32-5	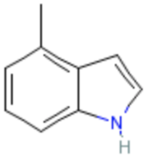	C_9H_9N	Y	N
59.177	13-Docosenamide,(Z)-	35	337.334	000112-84-5	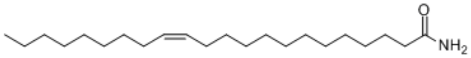	$C_{22}H_{43}NO$	Y	N
66.741	Benzo[h]quinoline,2,4-dimethyl-	45	207.105	000605-67-4	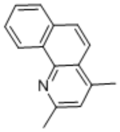	$C_{15}H_{13}N$	Y	N
				**Phenylpropanoid Derivatives/Benzenoids**				
10.977	Phenol	25	94.042	000108-95-2	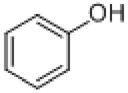	C_6H_6O	N	Y
14.506	Acetophenone	91	120.058	000098-86-2	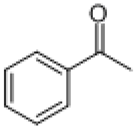	C_8H_8O	Y	Y

15.399	.alpha.-Ethyl-.alpha.-methylbenzylalcohol	72	150.104	001565-75-9	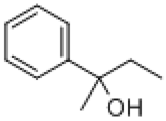	$C_{10}H_{14}O$	N	Y
15.400	dl-2-Phenyl-1,2-propanediol	78	152.084	004217-66-7	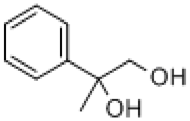	152.19	Y	Y
30.182	Phenol,2,4-bis(1,1-dimethylethyl)-	96	206.167	000096-76-4	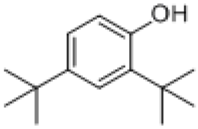	$C_{14}H_{22}O$	Y	Y
31.793	Benzene,1,4-dimethoxy-2-methyl-	70	152.084	024599-58-4	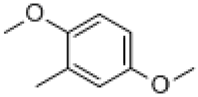	$C_{9}H_{12}O_{2}$	Y	N
34.429	Phenol,2-methoxy-4-propyl-	59	166.099	002785-87-7	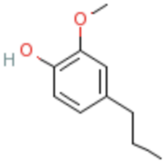	$C_{10}H_{14}O_{2}$	N	Y
38.581	Phenol,3,5-dimethoxy-	47	154.063	000500-99-2	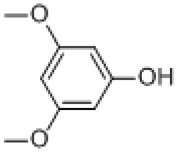	$C_{8}H_{10}O_{3}$	N	Y
38.317	3-Acridinol	43	195.068	007132-70-9	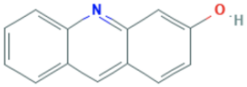	$C_{13}H_{9}NO$	Y	Y

38.600	Flopropione	38	182.058	002295-58-1	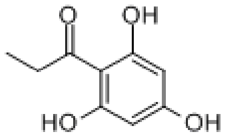	$C_9H_{10}O_4$	Y	Y
42.293	Phthalicacid,bis(7-methyloctyl)ester	59	418.308	020548-62-3	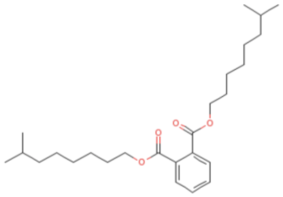	$C_{26}H_{42}O_4$	N	Y
49.754	Tiaprofenic acid	43	260.31	1000227-01-4	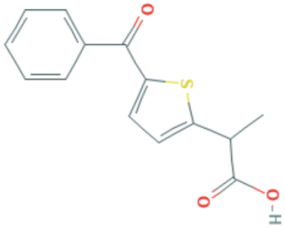	$C_{14}H_{12}O_3S$	N	Y
50.516	4,2-Cresoticacid,6-methoxy-,bimol.ester,methylester,4,6-dimethoxy-o-toluate	47	538.184	019314-74-0	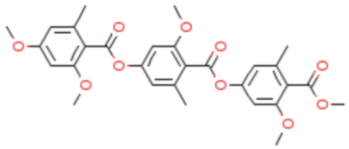	$C_{29}H_{30}O_{10}$	N	Y
52.207	Phenol,2,2'-methylenebis[6-(1,1-dimethylethyl)-4-methyl-	95	340.24	000119-47-1	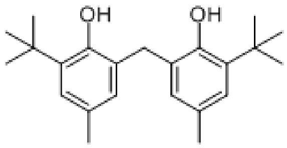	$C_{23}H_{32}O_2$	Y	Y
53.642	2,4-Difluorophenol	35	130.023	000367-27-1	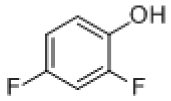	$C_6H_4F_2O$	Y	N

54.015	Indan,1-methyl-	47	132.094	000767-58-8	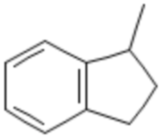	$C_{10}H_{12}$	Y	N
54.674	1,2-Benzenedicarboxylic acid, mono(2-ethylhexyl) ester	90	278.152	004376-20-9	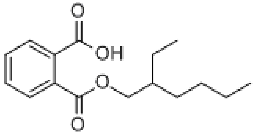	$C_{16}H_{22}O_4$	Y	Y
55.172	Phenol,4,4'-methylenebis[2,6-bis(1,1-dimethylethyl)-	55	424.334	000118-82-1	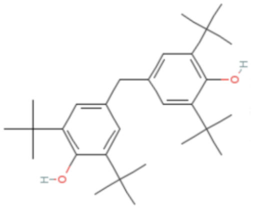	$C_{29}H_{44}O_2$	N	Y
57.757	Benzene,1,1'-(2-butene-1,4-diyl)bis-	45	208.125	013657-49-3	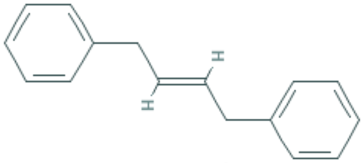	$C_{16}H_{16}$	Y	N
58.555	Terephthalicacid,di(2-ethylhexyl)ester	46	390.277	1000324-01-0	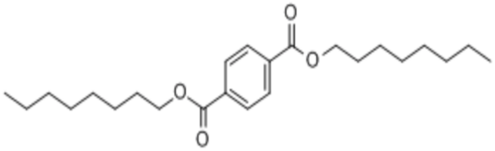	$C_{24}H_{38}O_4$	Y	N
59.404	1H-Indole,5-methyl-2-phenyl-	18	207.105	013228-36-9	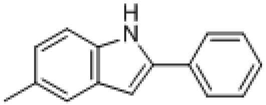	$C_{15}H_{13}N$	Y	N

62.004	Propiophenone,2'-(trimethylsiloxy)-	38	222.108	033342-87-9	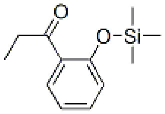	$C_{12}H_{18}O_2Si$	Y	N
62.590	Silane,trimethyl[5-methyl-2-(1-methylethyl)phenoxy]-	49	222.144	055012-80-1	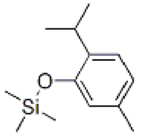	$C_{13}H_{22}OSi$	Y	N
65.592	Silane,1,4-phenylenebis[trimethyl-	53	222.126	013183-70-5	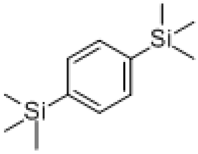	$C_{12}H_{22}Si_2$	Y	N
65.767	2,4,6-Cycloheptatrien-1-one,3,5-bis-trimethylsilyl-	50	250.121	1000161-21-8	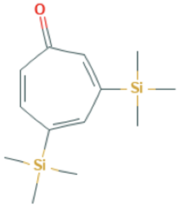	$C_{13}H_{22}OSi_2$	Y	N
66.880	(Phenylthio)acetic acid, 1-adamantylmethyl ester	25	316.15	1000299-95-7	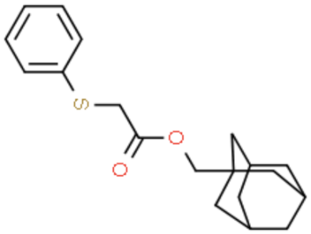	$C_{19}H_{24}O_2S$	N	Y
					**Triterpene**			
59.851	2,6,10,14,18,22-Tetracosahexaene,2,6,10,15,19,23-hexamethyl-,(all-E)-	99	410.391	000111-02-4	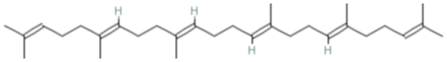	$C_{30}H_{50}$	Y	N

'Y' indicates the compound identified in the strain, 'N' indicates that the compound is not detected in the strain.

Table S2. Volatile metabolites assessed via GC-MS analysis in WT (A133 strain) and NT strains *in vivo* were analyzed with the Agilent ChemStation software. The chemical structure or the chemical structural formula was analyzed in pubchem database (https://pubchem.ncbi.nlm.nih.gov).

RT (min)	Volatile Compound	Qulity (%)	mz	CAS Number	Structure	Chemical Structural Formula	WT	NT
				**Fatty Acid Derived Volatiles**				
19.361	Ethanol,2-(2-butoxyethoxy)-	90	162.126	000112-34-5	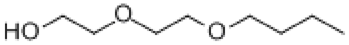	$C_8H_{18}O_3$	Y	Y
31.046	Sulfurous acid, isohexyl pentyl ester	50	236.145	1000309-14-0	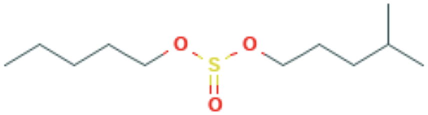	$C_{11}H_{24}O_3S$	Y	N
39.935	1,2-Benzenedicarboxylic acid, bis(2-methylpropyl) ester	78	278.152	000084-69-5	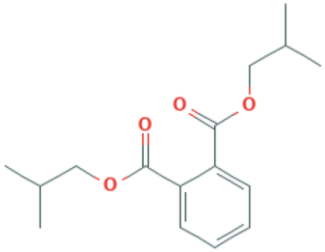	$C_{16}H_{22}O_4$	Y	N
41.663	Hexadecanoicacid,methylest er	95	270.256	000112-39-0	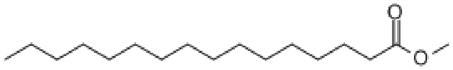	$C_{17}H_{34}O_2$	Y	Y
42.491	n-Hexadecanoicacid	99	256.24	000057-10-3	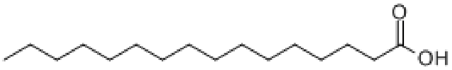	$C_{16}H_{32}O_2$	Y	Y
43.318	Hexadecanoicacid,ethylester	96	284.272	000628-97-7	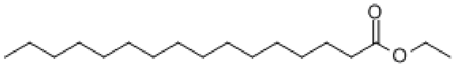	$C_{18}H_{36}O_2$	Y	Y

46.43	9,12-Octadecadienoicacid(Z,Z)-	99	280.24	000060-33-3	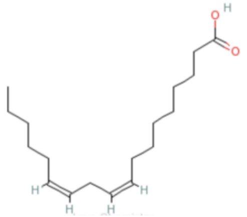	$C_{18}H_{32}O_2$	Y	Y
46.576	cis-13-Octadecenoicacid	98	282.256	013126-39-1	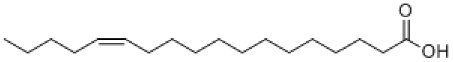	$C_{18}H_{34}O_2$	Y	Y
47.111	Linoleicacidethylester	99	308.272	000544-35-4	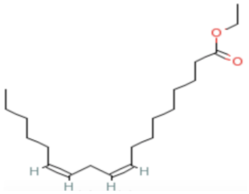	$C_{20}H_{36}O_2$	Y	Y
47.265	EthylOleate	99	310.287	000111-62-6	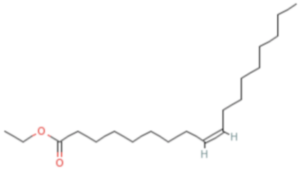	$C_{20}H_{38}O_2$	Y	Y
47.433	Hexadecanamide	99	255.256	000629-54-9	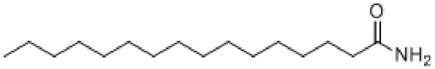	$C_{16}H_{33}NO$	N	Y
47.645	Ethanol, 2-(9-octadecenyloxy)-, (Z)-	70	312.303	005353-25-3	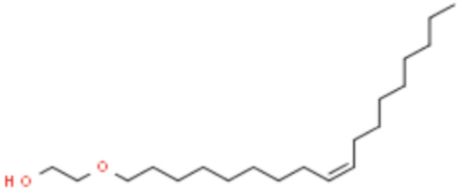	$C_{20}H_{38}$	Y	N

47.66	Oleyl alcohol , acetate	60	310.287	1000352-67-9	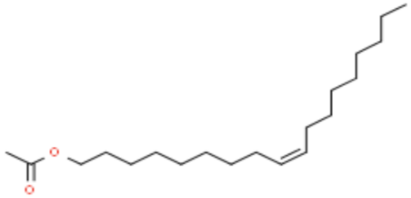	$C_{20}H_{38}$	N	Y
47.894	Octadecanoic acid, ethyl ester	95	312.303	000111-61-5	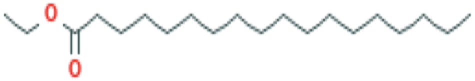	$C_{20}H_{40}O_2$	N	Y
49.754	Propanedioic acid, ethyl-, bis(1-methylpropyl) ester	47	244.167	057983-52-5	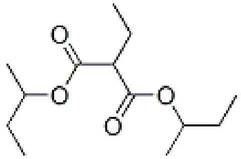	$C_{13}H_{24}O_4$	N	Y
50.728	4-Methyl-Z-4-hexadecen-1-ol	38	254.261	1000130-89-0	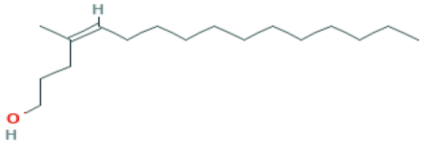	$C_{17}H_{34}O$	Y	Y
51.116	n-Propyl 9,12-octadecadienoate	49	322.287	1000336-77-8	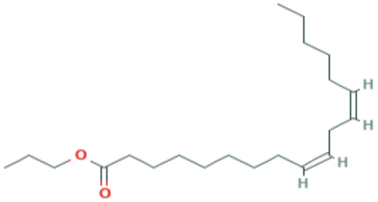	$C_{21}H_{38}O_2$	N	Y
51.116	cis-7-Oxabicyclo[4.3.0]nonan-8-one	47	140.084	024871-12-3	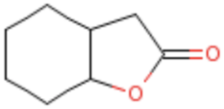	$C_8H_{12}O_2$	Y	Y

51.951	Hexanedioicacid,bis(2-ethylhexyl)ester	93	370.308	000103-23-1	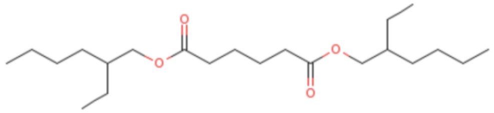	$C_{22}H_{42}O_4$	Y	Y
54.411	Hexadecanoicacid,2-hydroxy-1-(hydroxymethyl)ethylester	43	330.277	023470-00-0	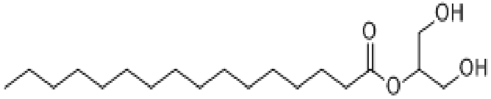	$C_{19}H_{38}O_4$	Y	N
66.448	Ergosterol	74	396.339	000057-87-4	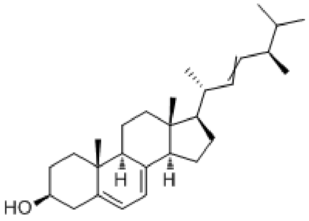	$C_{28}H_{44}O$	Y	Y
				**Saccharide Derived Volatiles**				
34.781	Benzo[b]tetrahydrofuran-3-one,5,6-dihydroxy-	59	166.027	014771-00-7	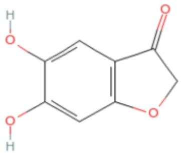	$C_8H_6O_4$	Y	Y
41.253	D-Glucoside	50	194.18	000097-30-3	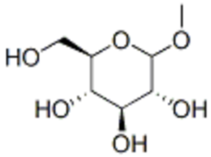	$C_7H_{14}O_6$	Y	Y
66.873	Acetamide,2-chloro-N-(3-cyano-4,6-dihydro-4,4,6,6-tetramethylthieno[2,3-c]furan-2-yl)-	4	298.054	1000350-39-4	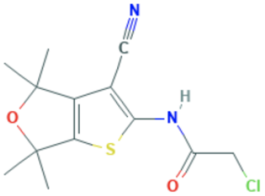	$C_{13}H_{15}ClN_2O_2S$	Y	N

Amino Acids Derived Volatiles

29.333	N-Methyl-N-methoxy-5,6,7,8-tetrahydro-1-naphtamide	64	219.126	185957-97-5	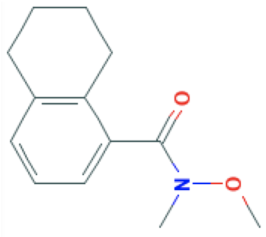	$C_{13}H_{17}NO_2$	Y	N
29.655	Caulophylline	87	204.27	000486-86-2	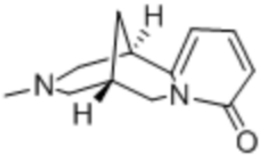	$C_{12}H_{16}N_2O$	Y	N
34.986	Nepetalactone	46	166.099	000490-10-8	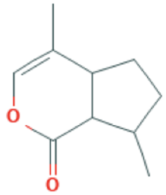	$C_{10}H_{14}O_2$	Y	N
35.784	Sulfurous acid, hexyl octyl ester	53	278.192	1000309-13-0	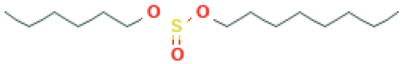	$C_{14}H_{30}O_3S$	N	Y
38.31	Dibenz[b,f]][1,4]oxazepine	43	195.068	000257-07-8	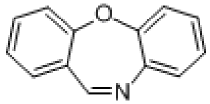	$C_{13}H_9NO$	Y	N
38.617	N,N'-Diacetyl-2-nitro-p-phenylenediamine	53	237.075	005345-53-9	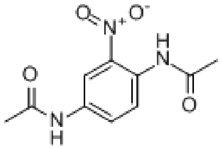	$C_{10}H_{11}N_3O_4$	Y	N

40.111	Isoelemicin	64	208.25	1000323-76-1	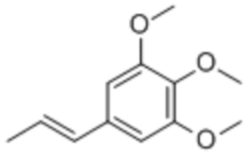	$C_{12}H_{16}O_3$	N	Y
41.004	7,9-Di-tert-butyl-1-oxaspiro(4,5)deca-6,9-diene-2,8-dione	99	276.173	082304-66-3	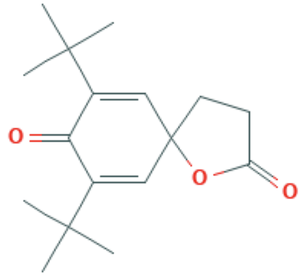	$C_{17}H_{24}O_3$	Y	Y
41.202	9-Aminoacridine	50	194.23	097825-91-7	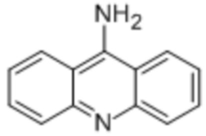	$C_{13}H_{10}N_2$	N	Y
41.473	Pyrrolo[1,2-a]pyrazine-1,4-dione,hexahydro-3-(2-methylpropyl)-	53	210.137	005654-86-4	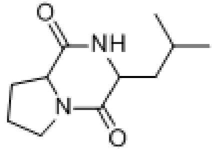	$C_{11}H_{18}N_2O_2$	Y	N
42.3	2,8-Dihydroxyadenine	58	167.12	30377-37-8	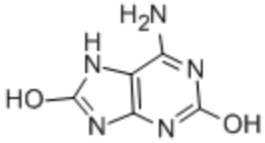	$C_5H_5N_5O_2$	Y	Y

46.21	2-Pyrrolidinone	18	85.053	000616-45-5	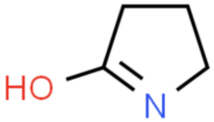	$C_{25}H_{52}$	Y	N
49.754	Pentanoic acid, 5-(acetylamino)-4-oxo-, methyl ester	43	187.084	093393-93-2	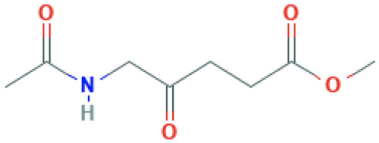	$C_8H_{13}NO_4$	Y	Y
51.277	9-Octadecenamide,(Z)-	99	281.272	000301-02-0	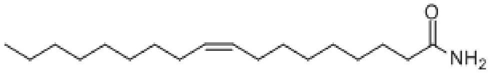	$C_{18}H_{35}NO$	Y	Y
51.826	Octadecanamide	94	283.288	000124-26-5	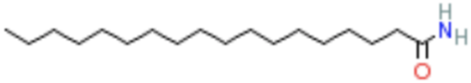	$C_{18}H_{37}NO$	Y	Y
53.642	1H-Indole,3-methyl-	47	131.073	000083-34-1	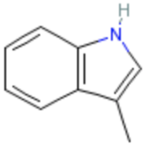	C_9H_9N	Y	Y
54.037	Quinoline,1,2,3,4-tetrahydro-1-((2-phenylcyclopropyl)sulfonyl)-,trans-	43	313.114	017299-24-0	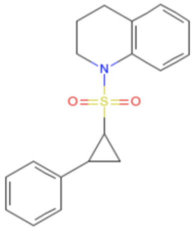	$C_{18}H_{19}NO_2S$	N	Y
57.449	1H-Indole, 6-methyl-	43	131.073	003420-02-8	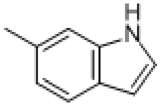	C_9H_9N	N	Y
				**Phenylpropanoid Derivatives/Benzenoids**				

14.506	Acetophenone	91	120.058	000098-86-2	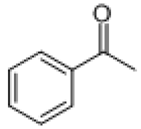	C_8H_8O	Y	Y
15.400	dl-2-Phenyl-1,2-propanediol	78	152.084	004217-66-7	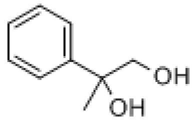	152.19	Y	Y
28.330	Dimethylphthalate	91	194.058	000131-11-3	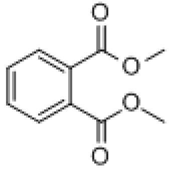	$C_{10}H_{10}O_4$	Y	Y
30.182	Phenol,2,4-bis(1,1-dimethylethyl)-	96	206.167	000096-76-4	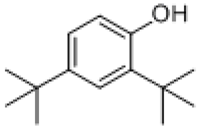	$C_{14}H_{22}O$	Y	Y
31.91	Benzaldehyde,-2-propoxy,-5-methoxy	60	194.094	1000222-96-5	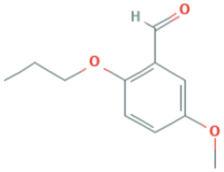	$C_{11}H_{14}O_3$	N	Y
35.22	Durohydroquinone	72	166.099	000527-18-4	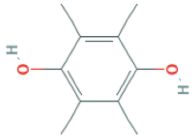	$C_{10}H_{14}O_2$	N	Y
44.307	9H-Fluoren-9-ol,9-butyl-	56	238.136	005806-10-0	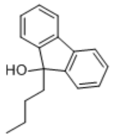	$C_{17}H_{18}O$	N	Y

45.441	1,4-Naphthoquinone,6-ethyl-2,5-dihydroxy-	25	218.058	013378-87-5	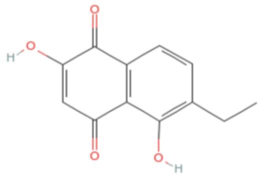	$C_{12}H_{10}O_4$	Y	N
52.207	Phenol,2,2'-methylenebis[6-(1,1-dimethylethyl)-4-methyl-	94	340.24	000119-47-1	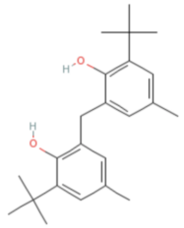	$C_{23}H_{32}O_2$	Y	Y
54.674	1,2-Benzenedicarboxylicacid,mono(2-ethylhexyl)ester	91	278.152	004376-20-9	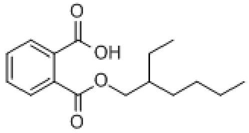	$C_{16}H_{22}O_4$	Y	Y
57.449	Naphthalene,1,2,3,4-tetrahydro-1-methoxy-	43	162.104	001008-18-0	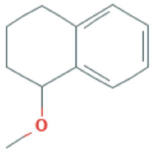	$C_{11}H_{14}O$	Y	Y
				**Triterpene**				
59.851	2,6,10,14,18,22-Tetracosahexaene,2,6,10,15,19,23-hexamethyl-,(all-E)-	99	410.391	000111-02-4	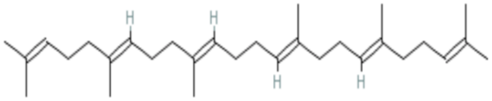	$C_{30}H_{50}$	Y	Y

'Y' indicates the compound identified in the strain, 'N' indicates that the compound is not detected in the strain.
